# Pharmacological evidence for the possible involvement of the NMDA receptor pathway in the anticonvulsant effect of tramadol in mice

**DOI:** 10.3934/Neuroscience.2022024

**Published:** 2022-11-10

**Authors:** Mazyar Zahir, Amir Rashidian, Mohsen Hoseini, Reyhaneh Akbarian, Mohsen Chamanara

**Affiliations:** 1 Department of Pharmacology, School of Medicine, Tehran University of Medical Sciences, Tehran, Iran; 2 Experimental Medicine Research Center, Tehran University of Medical Sciences, Tehran, Iran; 3 Department of Pharmacology, School of Medicine, Aja University of Medical Sciences, P.O. Box 1411718541, Tehran, Iran

**Keywords:** Anticonvulsants, mice, N-Methylaspartate, seizures, tramadol

## Abstract

**Background:**

Previous studies have shown controversial results regarding the pro- or anticonvulsant effects of tramadol. Additionally, the underlying mechanism of seizure induction or alleviation by tramadol has not been fully understood. In the current study, the effects of tramadol on pentylenetetrazole (PTZ)-induced seizure and the possible involvement of the N-methyl-D-aspartate (NMDA) pathway were assessed in mice.

**Methods:**

Male Naval Medical Research Institute (NMRI) mice were treated with intravenous infusion of PTZ in order to induce clonic seizures and determine seizure threshold. Tramadol was injected intraperitoneally (0.1–150 mg/kg) 30 minutes prior to elicitation of seizures. The possible effects of intraperitoneal injections of NMDA receptor antagonists, ketamine (0.5 mg/kg) and MK-801 (0.5 mg/kg) on the anticonvulsant property of tramadol were investigated subsequently.

**Results:**

Tramadol (1–100 mg/kg) increased PTZ-induced seizure threshold in a dose-dependent, time-independent manner, with optimal anticonvulsant effect at a dose of 100 mg/kg. Acute administration of either ketamine (0.5 mg/kg) or MK-801 (0.5 mg/kg) potentiated the anticonvulsant effect of a subeffective dose of tramadol (0.3 mg/kg).

**Conclusion:**

These results suggest a possible role of the NMDA pathway in the anticonvulsant effect of tramadol.

## 1. Introduction

Tramadol is an opioid analgesic, commonly used to alleviate moderate to severe pain in clinical settings [1]. While primarily recognized as a partial agonist of µ opioid receptors, it also effectively inhibits serotonin and norepinephrine uptake in the central nervous system [2]. Additionally, it has been postulated that tramadol may exert analgesic effects through regulation of various ion channels [3]. Despite not having the typical opioid side effects (e.g., respiratory depression, constipation, altered mental status), seizurogenic adverse effects have been reported with high dose or long-term therapy with tramadol in clinical studies [1],[4]. Paradoxically, animal studies have demonstrated an anticonvulsant property for low dose tramadol in rodents [5]–[7]. Gamma-aminobutyric acid (GABA), N-methyl-D-aspartate (NMDA) and kappa opioid pathways have been established as prominent role players in exerting the anticonvulsant effects of tramadol in maximal electroshock (MES) seizure model in mice [7]. Other studies have revealed the significant contribution of the nitric oxide (NO) pathway to the anticonvulsant properties of tramadol in pentylenetetrazole (PTZ)-induced seizures [6]. Additionally, the crucial role of the NMDA pathway in seizure and epileptogenesis has been established [8],[9]. Despite the aforementioned evidence, the possible contribution of the NMDA pathway to the anticonvulsant property of tramadol in PTZ-induced seizure model has never been investigated previously.

NMDA receptors are ligand-gated ion channels mostly expressed in neurons and activated by the simultaneous binding of glutamate and glycine, or their synthetic analogs. This leads to an influx of calcium into the cell, which in turn activates molecular signaling pathways [10],[11]. The pivotal role of the NMDA pathway in various neurologic and psychiatric disorders (e.g., Alzheimer's disease, schizophrenia, depression, seizure) has been discovered previously [7],[12]–[14]. Moreover, former studies have indicated that NMDA receptors are involved in the antinociceptive, antidepressant and memory enhancing effects of tramadol [14]–[16]. Considering the dual aggravating and attenuating features of tramadol on convulsions and its unspecified mechanism of action, we hypothesized that tramadol may produce its pro- or anticonvulsant effects via the NMDA pathway. Consequently, the current study was designed and conducted to investigate the possible effects of tramadol and probable role of NMDA receptors (NMDARs) on the threshold of PTZ-induced seizure.

## 2. Materials and methods

### 2.1. Animals and housing conditions

Male NMRI mice were purchased from Pasteur Institute (Iran) and were used throughout this research. The total number of mice used in our investigation was 132, all weighing between 25 and 30 grams. All mice were housed in groups of six per cage on a standard 12 hour light/dark circadian cycle in a temperature-controlled room (25 ± 1 °C). Food and water were freely accessed by all animals. All experiments took place between 9:00 AM and 1:00 PM. Institutional guidelines for animal care, handling and use were observed throughout the study. Each treatment group consisted of 6 subjects, and each animal was used only once and then sacrificed humanely by applying firm pressure at the base of the skull while simultaneously pulling its tail backwards [17].

### 2.2. Drugs and chemicals

The µ-opioid receptor agonist tramadol was purchased from Aboureihan, Tehran, Iran. Pentylenetetrazole; ketamine, a noncompetitive NMDAR antagonist; and MK-801, an uncompetitive NMDAR antagonist, were all bought from Sigma, St. Louis, MO, USA. Physiological normal saline solution was used to dilute all drugs to a concentration at which the desired doses could be administered in a constant volume of 5 ml/kg body weight. All drugs were injected intraperitoneally, except for PTZ, which was administered intravenously via the tail vein. All drug preparations were done immediately prior to the experiment, and proper vehicle controls were used in each experiment.

### 2.3. Determination of seizure threshold

A mouse restrainer was used to restrain mice with access to their tails. A 30-gauge butterfly needle was inserted into the tail vein and fixed to the tail with a small piece of adhesive tape, without restricting mouse movement. In order to induce seizure, PTZ (5 mg/ml) was infused continuously with a constant rate of 1 ml/minute until forelimb clonus succeeded by full body clonus was observed. The minimum dose of PTZ (mg/kg mouse weight) required to induce generalized convulsing movements was defined as the index of seizure threshold. As such, seizure threshold is influenced by the dose and time of PTZ administration [18],[19].

### 2.4. Treatment

At the first step, the time course in which tramadol would exert its maximal pro- or anticonvulsant effect was determined. Animals received tramadol (1 mg/kg of body weight) 30, 60 and 120 minutes before evaluation of PTZ-induced seizure threshold. Control subjects were treated with normal saline (5 ml/kg) simultaneously [6].

At the second step, different doses of tramadol (0.1, 0.3, 1, 3, 50, 100 and 150 mg/kg) were injected intraperitoneally in an effort to discover the dosage capable of eliciting the most notable pro- or anticonvulsant effect. Tramadol administration took place 30 minutes prior to PTZ injection. The doses and time lag of tramadol injection were chosen based on previous studies [5],[6]. Normal saline (5 ml/kg) was administered as a corresponding vehicle, 15 minutes before tramadol.

In the last phase, in order to assess the possible role of the NMDA pathway in the pro- or anticonvulsant effect of tramadol, NMDAR antagonists were co-administered with a sub-effective dose of tramadol (0.3 mg/kg). This dosage of tramadol is considered sub-effective, since it has failed to exert any anticonvulsant effect when injected alone [5], [6]. Ketamine (0.5 mg/kg) or MK-801 (0.5 mg/kg) was administered 15 minutes before either normal saline (5ml/kg) or tramadol (0.3 mg/kg) and 45 minutes before induction of seizure with PTZ [20].

### 2.5. Statistical analysis

All results are expressed as means ± SEM of PTZ dose (mg/kg) of 6 mice and analyzed using GraphPad Prism 9 statistical software (GraphPad Software Inc., La Jolla, California, USA). Two-way analysis of variance (ANOVA) and Bonferroni's test were used to analyze the data obtained from our time-course experiment. The main rationale behind using two-way ANOVA for our time-course experiment was evaluating the individual effects of time and treatment and also their interaction on changing the seizure threshold. All other investigational data were analyzed using one-way ANOVAs followed by Bonferroni's post hoc tests. P < 0.05 was considered statistically significant.

### 2.6. Ethics

Our study was in accordance with the National Institutes of Health (NIH) Guidelines for the Care and Use of Laboratory Animals (HHS publication 85-23, 1985) and legislation for the protection of animals used for scientific purposes (Directive 2010/63/EU). Additionally, this study was ethically reviewed and approved by Tehran University of Medical Sciences (TUMS) ethical review board.

## 3. Results

### 3.1. Effect of different times of tramadol administration on seizure threshold

**Figure 1.**
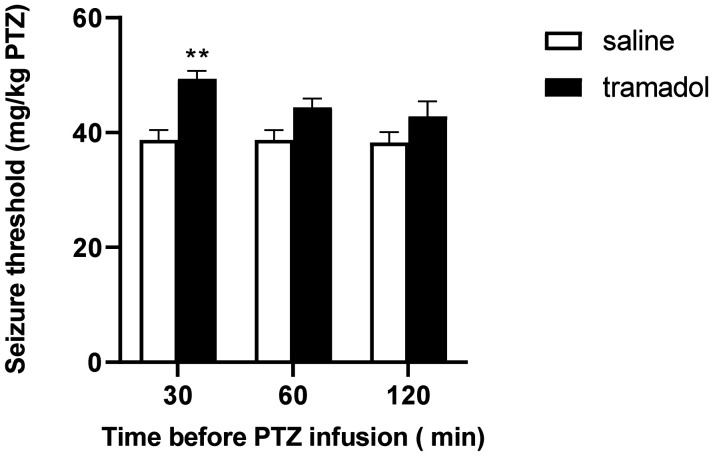
Effect of timing of tramadol (1 mg/kg) injection on PTZ-induced seizure threshold in mice. All data are calculated and expressed as means ± SEM of 6 subjects. “Seizure threshold” in the labeling on the Y axis is the shortened form of “seizure onset threshold.” Statistical analysis was performed by two-way ANOVA followed by Bonferroni's test. ** P < 0.01 compared with saline-treated control groups.

The results displayed in Figure 1 show that although tramadol (1 mg/kg) augmented seizure threshold in all time intervals, this increase was only significant in the mice which were treated with tramadol 30 minutes prior to seizure induction. Interestingly, our two-way ANOVA revealed that this increment is only owing to the extremely significant effect of tramadol treatment (*F*[1, 30] = 21.34, P < 0.0001), since no statistically significant effect was seen for the time of tramadol administration (*F*[2, 30] = 1.926, P=0.1633) or for the time-treatment interaction (*F*[2, 30] = 1.557, P = 0.2274). No proconvulsant effect was observed in any time interval.

### 3.2. Effect of different doses of tramadol on seizure threshold

**Figure 2.**
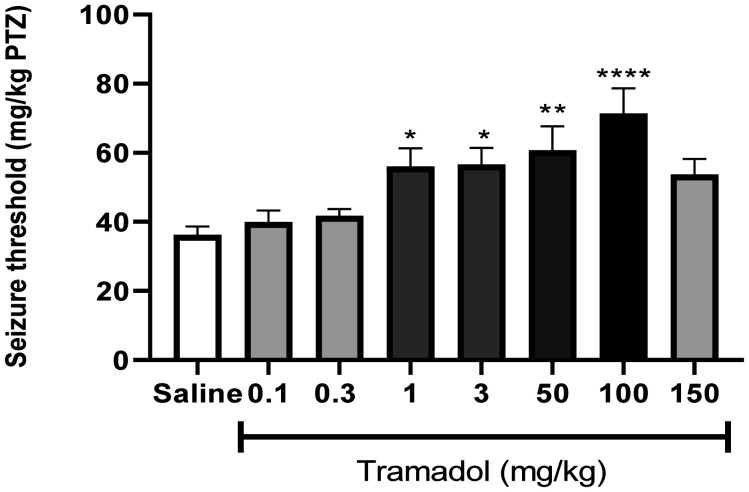
Effects of various doses of tramadol (0.1, 0.3, 1, 3, 50, 100 and 150 mg/kg) on PTZ-induced seizure threshold in mice. Data are expressed as means ± SEM of 6 mice. “Seizure threshold” in the labeling on the Y axis is the shortened form of “seizure onset threshold.” Statistical analysis was performed by one-way ANOVA followed by Bonferroni's post hoc test. * P < 0.05, **P < 0.01 and **** P < 0.0001 compared with saline-treated group.

As illustrated in Figure 2, acute administration of tramadol (1, 3, 50 and 100 mg/kg) significantly increased seizure threshold when injected 30 minutes prior to PTZ (*F*[7, 40] = 5.736, P < 0.001). A tramadol dose of 100 mg/kg was associated with the highest increase of seizure threshold (P < 0.0001). Additionally, very low (0.1 and 0.3 mg/kg) and very high (150 mg/kg) doses of tramadol were incapable of enhancing seizure threshold significantly (P > 0.05). However, no proconvulsant effect was seen in the range of doses used in our study (0.1–150 mg/kg). Notably, none of the tramadol-treated subjects showed any sign of intoxication (e.g., restlessness, unsteady gait, reduced spontaneous activity, and slight cyanosis) in the course of this experiment.

### 3.3. Involvement of NMDA receptors in the anticonvulsant effects of tramadol

**Figure 3.**
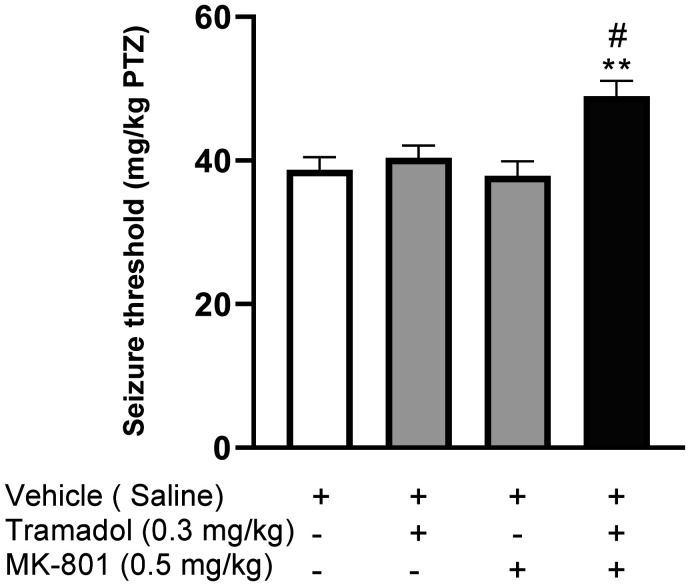
Effects of tramadol (0.3 mg/kg), MK-801 (0.5 mg/kg) and their combination on PTZ-induced seizure threshold. All data are expressed as means ± SEM of 6 mice. “Seizure threshold” in the labeling on the Y axis is the shortened form of “seizure onset threshold.” Statistical analysis was performed by one-way ANOVA followed by Bonferroni's post hoc test. **P < 0.01 compared with vehicle-treated control group. # P < 0.05 compared with tramadol-treated group.

**Figure 4.**
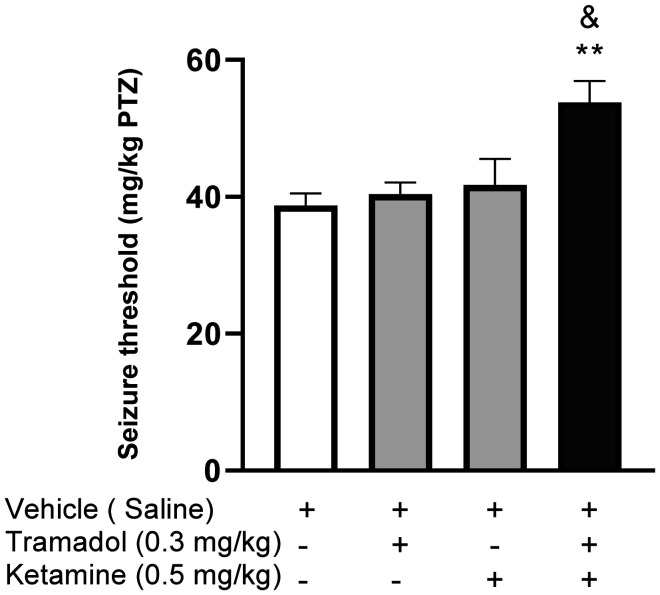
Effects of tramadol (0.3 mg/kg), ketamine (0.5 mg/kg) and their combination on PTZ-induced seizure threshold. All data are expressed as means ± SEM of 6 mice. “Seizure threshold” in the labeling on the Y axis is the shortened form of “seizure onset threshold.” Statistical analysis was performed by one-way ANOVA followed by Bonferroni's post hoc test. **P < 0.01 compared with saline-treated group. & P < 0.05 compared with tramadol-treated group.

As demonstrated in Figure 3, acute administration of MK-801 (0.5 mg/kg) did not increase the PTZ-induced seizure threshold (P = 0.9882); however, co-administration of MK-801 (0.5 mg/kg) and a subeffective dose of tramadol (0.3 mg/kg) significantly augmented seizure threshold in comparison with a saline-treated control group (*F*[3, 20] = 7.211, P < 0.01). Similarly, although ketamine (0.5 mg/kg) failed to considerably increase seizure threshold when injected alone (p = 0.8695), its co-administration with the same subeffective dose of tramadol (0.3mg/kg) significantly raised seizure threshold in comparison with the vehicle-treated control group (*F*[3, 20] = 6.255, P < 0.01, Figure 4). Altogether, these findings indicated that NMDAR inhibitors can augment tramadol effects.

## 4. Discussion

Tramadol, a low-potency µ opioid receptor agonist, was first introduced as an effective analgesic; however, shortly after its distribution, a seizurogenic side effect was reported at recommended doses among patients [4]. According to Centers for Disease Control and Prevention (CDC) Guidelines, the maximum approved daily dosage for tramadol is 400 mg (approximately 5 mg/kg) [21]. Interestingly, former animal experiments have demonstrated that seizures only occur in very high doses (30–50 mg/kg); and conversely, they have suggested an anticonvulsant effect for lower doses (1–10 mg/kg) of tramadol in PTZ-induced seizure, maximal electrical shock and kindled rats [5]–[7]. These paradoxical pro- or anticonvulsant effects probably depend on dose, route of administration and the animal model being studied.

According to our results, tramadol is capable of raising seizure threshold significantly in a wide spectrum of doses, ranging from 1 to 100 mg/kg, with a maximal antiepileptic effect at 100 mg/kg. This finding is in opposition with a former study which stated that tramadol exerts a seizurogenic effect at a dose of 50 mg/kg. This discrepancy is probably due to the different dose and route of PTZ administration (80 mg/kg, intraperitoneal injection) in that study [22]. Our results also revealed that both very low (0.1 and 0.3 mg/kg) and very high (150 mg/kg) doses are incapable of changing seizure threshold significantly. Furthermore, no signs of neurologic adverse effects were observed in the dose spectrum of our experiment. This finding was notable since it elucidated that, contrary to previous reports, administration of tramadol in doses much higher than 50 mg/kg (up to 150 mg/kg) is not associated with neurotoxicity [23].

Contrary to a previous study, our time course experiment failed to show any association between tramadol's anticonvulsant effect and its time of administration. This inconsistency is probably due to different statistical methods being utilized in our study (two-way ANOVA) vs. their study (one-way ANOVA) [6]. However, in order to precisely assess the possible effects of time and treatment and their interaction on seizure threshold, two-way ANOVA is the superior statistical method [24].

Previous studies have been mostly focused on the proconvulsant effects of tramadol and proposed various signaling pathways (e.g., opioid, serotonin, norepinephrine, GABA, dopamine and adenosine) as possible contributors in this regard [22],[25]. Surprisingly, there is a staggering scarcity of evidence on the possible interaction between the NMDA pathway and tramadol.

NMDA receptors are abundantly expressed in the central nervous system (CNS). Activation of these unique ligand-gated Ca^2+^ channels requires the simultaneous binding of glutamate and glycine, and release of Mg^2+^ blockade. The consecutive increase in permeability and sudden influx of Ca^2+^ triggers multiple downstream signaling pathways [10],[11],[26]. NMDARs have been formerly implicated in a wide variety of neurological and psychiatric pathologies. They have been shown to play a critical role in the pathophysiology of traumatic brain injury, ischemic stroke, schizophrenia, mood disorders, Alzheimer's disease, and epilepsy [12]–[14],[26]. Additionally, some recent studies have nominated NMDA blockade as a possible mechanism of action of newly proposed antiepileptic drugs [27],[28].

Earlier studies have confirmed that pretreatment with NMDAR antagonists (ketamine and MK-801) can effectively increase seizure threshold in mice prone to seizure due to acute administration of a µ-opioid agonist, methadone [29]. Accordingly, we used different NMDAR antagonists, MK-801 and ketamine, in order to evaluate the probable contribution of glutamatergic NMDA receptors to the anticonvulsant effect of tramadol. Our data showed for the first time that injection of either ketamine or MK-801 potentiated the anticonvulsant effect of a subeffective dose of tramadol (0.3 mg/kg) in PTZ-induced seizure. In accordance with our results, NMDA pathway was previously postulated as a possible role player in enhancing the anticonvulsant effect of tramadol in MES-induced seizure [7]. Conclusively, it can be deduced that there is an interlaced relation between opioid and NMDA systems in elicitation of tramadol's anticonvulsant effect. Moreover, previous studies have demonstrated the contribution of the NMDA pathway to the painkilling, antidepressant and memory enhancing properties of tramadol, with the nitric oxide (NO) pathway being the assumed linking mechanism between tramadol and NMDARs [16],[30]. Consequently, it is presumable that NMDAR antagonists may contribute to the anticonvulsant effects of tramadol through the NO pathway. An alternative explanation is the possible pharmacokinetic interaction between NMDAR antagonists and tramadol, possibly affecting the elimination of tramadol and thus augmenting its anticonvulsant effects [15]. Subsequently, additional investigation must be warranted in this regard to fully elucidate the underlying mechanisms.

It has to be noted that the main limitations of our study were an inability to evaluate the effect of NMDA agonists and NO pathway inducers and inhibitors on the anticonvulsant effect of tramadol due to funding shortage.

## 5. Conclusion

Evidence extracted from our study delineates the possible role of the NMDA pathway in the anticonvulsant effect of tramadol for the first time. It was shown that tramadol can effectively augment seizure threshold in a dose-dependent, time independent manner. This enhancement takes place in a wide range of doses without causing any side effects. It is noteworthy that no proconvulsant effect was observed in the range of doses used in our study. Finally, further studies with various combinations of NMDA and NO pathway ligands and determination of molecular components, such as nitric oxide measurement, must take place in order to discover the detailed mechanisms and biochemical changes in the CNS in this convulsive model.
